# COVID‐19 vaccine‐induced vasculitis in a patient with sarcoidosis: A case report

**DOI:** 10.1002/ccr3.6501

**Published:** 2022-12-02

**Authors:** Ehsan Rahmanian, Majid Alikhani, Maryam Loghman, Sara Beikmohamadi Hezaveh, Saba Zangeneh, Reza Shahriarirad, Seyedeh Tahereh Faezi, Mohammad Nejadhosseinian

**Affiliations:** ^1^ Department of Rheumatology Hormozgan University of Medical Sciences Bandar Abbas Iran; ^2^ Department of Internal Medicine, School of Medicine, Rheumatology Research Center, Shariati Hospital Tehran University of Medical Sciences Tehran Iran; ^3^ Resident of Neurology, Department of Neurology, Shariati Hospital Tehran University of Medical Sciences Tehran Iran; ^4^ School of Medicine Fasa University of Medical Sciences Shiraz Iran; ^5^ School of Medicine Shiraz University of Medical Sciences Shiraz Iran; ^6^ Thoracic and Vascular Surgery Research Center Shiraz University of Medical Sciences Shiraz Iran; ^7^ Joint Reconstruction Research Center Tehran University of Medical Sciences Tehran Iran; ^8^ Rheumatology Research Center Tehran University of Medical Sciences Tehran Iran

**Keywords:** adverse effects, cutaneous vasculitis, mononeuritis multiplex, sarcoidosis, SARS‐COV‐2, Sinopharm COVID‐19 vaccine

## Abstract

A 55‐year‐old lady with a nine‐year history of controlled sarcoidosis developed vasculitis after Sinopharm COVID‐19 vaccine (BBIBP‐ CorV). She was ultimately diagnosed with mononeuritis multiplex based on EMG‐NCV findings and administered methylprednisolone and cyclophosphamide pulse therapy for 5 days, and then continue with prednisolone and a monthly pulse of cyclophosphamide.

## Graphical abstract

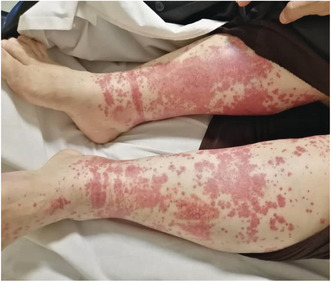

## 1 BACKGROUND

During the coronavirus disease 2019 (COVID‐19) pandemic, several vaccines have been generated by researchers and companies across the world such as Pfizer/BioNTech (BNT162b2) and Moderna (mRNA‐1273) COVID‐19 vaccines, mostly used in the United States, United Kingdom, and Europe,^1^, ^2^, ^3^ while CoronaVac and Sinophram are used particularly in Asia, the Middle East, and South America.^4^ Although the vaccines proved to be safe and beneficial, concerns about possible side effects, including cutaneous manifestations and more specifically, vasculitis have been debatable.

Small, medium, and large vessel vasculitis, all, have been seen in sarcoidosis. Although vasculitis is generally rarely reported in sarcoidosis, studies have shown that there is a suspicion of a link between vasculitis and sarcoidosis.^5^, ^6^, ^7^ Vasculitis following the COVID‐19 vaccines has also been reported.^8^, ^9^, ^10^, ^11^, ^12^ On this basis, we report a case of Sinopharm‐induced vasculitis in a patient with sarcoidosis.

## 2 CASE‐PRESENTATION

SSSThe patient is a 55‐year‐old woman with a nine‐year history of sarcoidosis with lower extremities arthritis and lung involvement, who received a daily dose of 200 mg hydroxychloroquine and 2.5 mg per day of prednisolone. Her sarcoidosis diagnosis was based on her shortness of breath and cough with joint symptoms, Erythema nodosum, and Hilar lymphadenopathy. The patient had bilateral ankle joint pain with inflammatory characteristics, such as morning stiffness of more than 30 min, along with swelling. Her disease was controlled and did not have any complications or recurrence.

**FIGURE 1:**
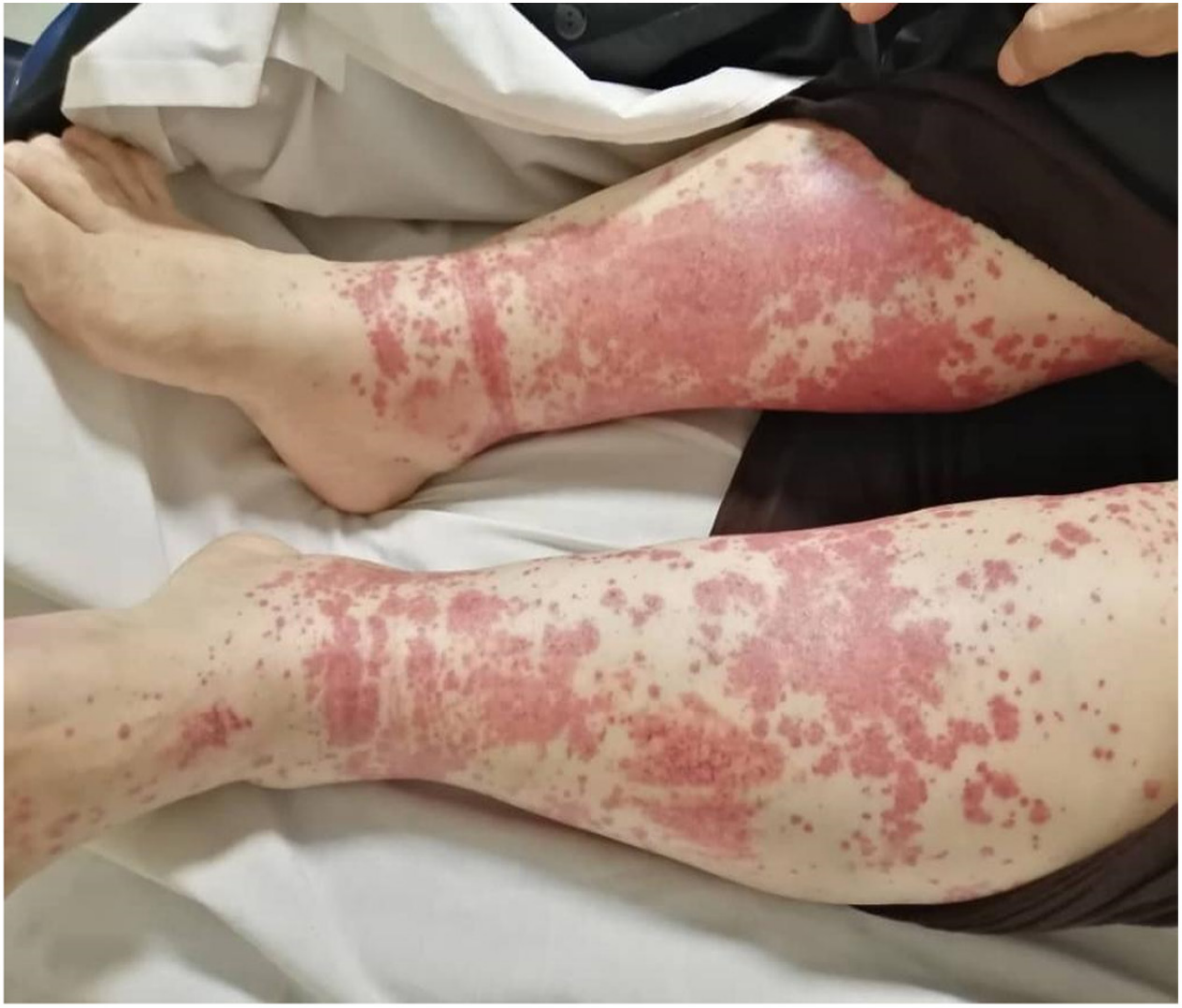
Symmetric distal limb swelling and palpable purpuric confluent macules in a 55‐year‐old female patient following the coronavirus disease vaccine

The patient first developed skin lesions in the form of palpable purpura 3 days following the first Sinopharm COVID‐19 vaccine (BBIBP‐ CorV). Palpable purpura initially appeared on the right foot, and subsequently, the left foot and spread to the flexor and extensor parts of both legs and thighs, without pruritus and pain (Figure 1). One week later, the patient developed paresthesia of the upper and lower limbs initially affecting the 4th and 5th of the right‐hand fingers and rapidly spreading to the left hand and both legs. After a week, paresthesia symptoms and then weakness and inability to grasp objects exaggerated and he was referred to the emergency department for evaluation with a chief complaint of extreme fatigue.

In physical examination, vital signs were normal without fever or respiratory distress, however, she had pitting edema (2+), which was accompanied by palpable and red color papules and purpuric skin lesions in the lower extremities that did not fade under pressure. There were no signs of lymphadenopathy or splenomegaly. Also, she had no history of recent weight loss and weighed 75 kg. The patient's neurological examination was sensory neuropathy in the distal right side upper extremity and superficial sensory disturbance in both legs, in addition to motor neuropathy in the right extremities. (Table 1) The ophthalmic evaluation was also unremarkable.

**TABLE 1:** Neurological examination of a 55‐year‐old female patient, known case of Sarcoidosis diagnosed with mononeuritis multiplex and cutaneous vasculitis

Motor evaluation of upper limbs from proximal to distal	
Shoulders	Both sides: normal force in abduction, adduction, flexion, extension, and rotations
Elbows	Both sides: normal force flexion and extension
Distal upper extremities	Right: a decrease in the abduction, adduction, and opposition muscle strength of the thumb (3/5), and also the abduction and adduction of the 5th digit, along with reduced power of handgrip. Left: normal
Sensory examination of the upper limbs	
Proximal	Normal without any significant findings
Distal	A decrease in the palm side sensation of the first to the third digit, and a slighter decrease in the fourth digit finger 4 in the palm. (Median nerve range)
Lower limbs from the proximal to the distal	
Hips	Both sides: normal without any significant findings in force examination and flexion, extension, abduction, adduction, and rotation movements.
Knee	Both sides: normal force
Distal lower extremity	Right side: decreased muscle force and disturbance in the dorsiflexion of the right foot. (3/5) Left side: normal without any significant findings
Lower extremity sensory examination	
Superficial	Decreased sensation in the s1 neural area, the presence of a plain that was evident in superficial touch. Sensory disturbance in the sural area and posterolateral and dorsal surface of both legs especially the right leg + light touch disturbance
Deep	Normal regarding position and vibration
Reflex	There was a decrease in the Achilles reflex of the right foot.
Ophthalmic evaluation	Normal

Laboratory tests revealed leukocytosis with neutrophil preference. Test results showed erythrocyte sedimentation rate (ESR) and C‐reactive protein (CRP) to be 89 and 178 (Normal <6), and a high level of D‐Dimer. Also, kidneys and liver function tests, albumin and total protein, cytoplasmic anti‐neutrophilic cytoplasmic antibodies (CANCA and Anti PR3), perinuclear anti‐neutrophil cytoplasmic antibodies (PANCA and anti‐MPO), cryoglobulins, antinuclear antibodies, anti‐ds DNA, C3, C4, serology for hepatitis B virus and hepatitis C and human immunodeficiency virus (HIV) were negative or normal. Moreover, urinalysis and stool examination did not have any findings in favor of infectious or renal diseases.

**FIGURE 2:**
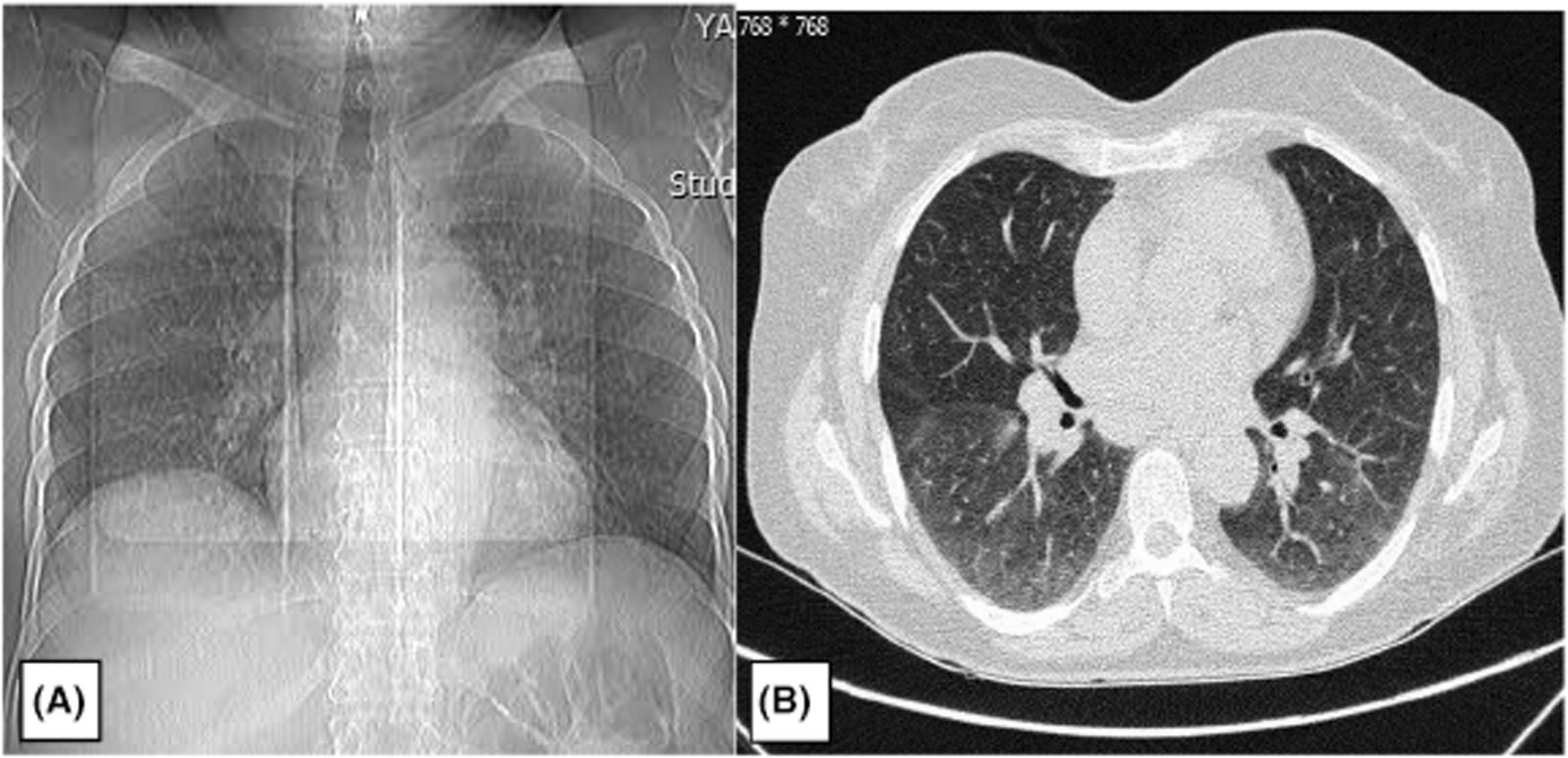
Chest X‐ray (A) and computed tomography scan (B) of a 55‐year‐old female patient with sarcoidosis, demonstrating bilateral hilar lymphadenopathy accompanied by faint ground‐glass opacities

Radiological evaluation with a chest X‐ray (CXR) and computed tomography scan showed bilateral hilar lymphadenopathy, which was also prevalent in her past CXR from her course of sarcoidosis disease, and without any noticeable change accompanied by faint ground‐glass opacities, which were seen in the lower lobes leading to a mosaic pattern with a nonspecific appearance (Figure 2). Electrocardiogram and echocardiography were normal with good ejection fraction and without any systolic or diastolic dysfunction. Electromyography‐nerve conduction velocity (EMG‐NCV) evaluation showed mono neuritis multiplex with chronic asymmetrical sensorimotor polyneuropathy. Additionally, the color doppler sonography (CDS) examination of both legs was normal.

Based on the patients' clinical symptoms and evaluations, along with the results of EMG‐NCV, and the recent COVID‐19 vaccine, a preliminary diagnosis of vasculitis was introduced, probably triggered by Sinopharm COVID‐19 vaccination. She was administered methylprednisolone succinate (1 g/daily for 5 days), followed by cyclophosphamide (1 g intravenous). She was discharged with oral prednisolone 1 mg/kg and recommended cyclophosphamide (1 g) monthly. After 1 week, we observed dramatic resolution and fading of her pale skin lesions and an increase in her muscle forces and after 2 weeks acute phase reactants decreased.

## 3 DISCUSSION

This study was intended to report vasculitis in a patient with sarcoidosis who received the Sinopharm COVID‐19 vaccine. Immunity, inflammation, and coagulation genes are part of the same (X) chromosome. Therefore, it may be suspected that viral interactions related to human genes can elicit an abnormal immune response in COVID‐19. Also, excessive antigen presence and the formation of relatively resistant soluble antigen–antibody immune complexes after facing SARS‐CoV‐2, according to Manzo's investigations may induce persistent inflammation in organs such as the skin.^13^ The risk of different types of vaccines in patients with diminished immune systems has been reported in previous studies.^14^, ^15^, ^16^, ^17^ Generally, our study puts the spotlight on the side effect of the COVID‐19 vaccination, specifically neurological involvement (mononeuritis multiplex) and cutaneous vasculitis, in a patient with sarcoidosis and can pave the way for future studies exploiting the adverse effect of vaccinations for this specific group of patients.

The COVID‐19 pandemic has caused a toll on many aspects of the healthcare system and also patients, and required immediate interventions and actions, some aiming to facilitate diagnosis,^18^, ^19^, ^20^, ^21^, ^22^, ^23^ some to improve treatment,^22^, ^24^, ^25^, ^26^, ^27^, ^28^, ^29^, ^30^ and some (such as vaccination and applied protocols) aim to subside the spread of the disease.^25^, ^29^, ^31^, ^32^, ^33^ In the process of emergency production of the COVID‐19 vaccine, immunocompromised patients were not included in the initial studies' study population.^14^ However, studies were conducted on the prevalence of COVID‐19 in sarcoidosis patients and an algorithm was designed for vaccination in these patients. This study ultimately states that the vaccination was found to be useful and safe for this group.^34^ Neurological involvement and cutaneous vasculitis were the main focus of our study. Neutrophilic inflammation mostly in the superficial cutaneous postcapillary venules is defined as cutaneous vasculitis,^35^ which in some cases can be secondary to infection, medication, and vaccination.^12^, ^36^ The onset and exacerbation of vasculitis symptoms have been reported during vaccinations against some viruses, such as influenza, hepatitis A and B, and Bacillus Calmette‐Guerin; however, cutaneous vasculitis following the COVID‐19 vaccine has been rarely reported in the literature.^37^, ^38^

Some cases related to various COVID‐19 vaccines' vasculitis including Bharat, Pfizer‐BioNTech, AstraZeneca, and Sinovac have been explored.^1^, ^2^, ^10^ Leukocytoclastic vasculitis in a 71‐year‐old woman without any noted allergy was diagnosed and announced 4 days after the second dose of the AstraZeneca vaccine by G. Fiorillo et al.^38^ Two other similar cases with the same diagnosis were reported after the first dose and the second dose of AstraZeneca by S. Sandhu et al.^16^ Cohen et al.^37^ reported leukocytoclastic vasculitis in a 46‐year‐old woman with a history of psoriasis and irritable bowel syndrome, after both doses of Pfizer‐BioNTech, affirmed vasculitis. An evaluation of 414 cases with cutaneous reactions after their first dose of the Pfizer‐BioNTech vaccine stated the frequency of vasculitis to be about 2.9%.^11^ A skin‐limited negative effect of Sinovac as Leukocytoclastic vasculitis in two young women without any known medical history has also been reported.^4^

Inactivated virus SARS‐CoV‐2 vaccine, Sinopharm, caused cutaneous vasculitis in our case. After performing laboratory tests, an increase in inflammatory markers was witnessed. Vaccine‐induced vasculitis suggests that inflammatory markers were reduced after 2 weeks with methylprednisolone and cyclophosphamide. Also, no further sarcoidosis‐related features such as lung involvement were observed in our patient.

To sum up our case, the control of sarcoidosis in the course of the disease and the absence of new symptoms based on the activation of sarcoidosis (arthritis, erythema nodosum, and cough) and the normality of paraclinical studies suggest the activity of sarcoidosis (eye examination. 24‐hour urinary calcium electrocardiogram and echocardiography) and the development of skin symptoms suggestive of leukocytoclastic vasculitis (although not performed to definitively confirm skin biopsy) and multiple mononuritis, all and all made us conclude that our patient is a case of vasculitis following the COVID‐19 vaccination. According to literature, vaccination is not contraindicated in immunocompromised patients and it can be performed in patients with sarcoidosis based on provided guidelines and consultations with rheumatologists, however, cutaneous manifestations of the vaccine such as vasculitis should not go unnoticed.^4^, ^9^

## 4 CONCLUSION

Vaccination, as a factor alone or in combination with disease agents, can cause new or aggravate pre‐existing symptoms, such as vasculitis among sarcoidosis patients. Although mononeuritis multiplex and cutaneous vasculitis have been reported among other vaccines. However, some precautions are demanded, these complications among COVID‐19 vaccines have been rarely reported and should especially be taken into consideration among patients prone to rheumatological diseases. Apart from the negative effect of the proposed vaccine, studies demonstrated that the advantages of the vaccination outweigh the disadvantages.

## AUTHOR CONTRIBUTIONS

E.R, M.A., M.L, and S.T.F made the disease diagnosis and carried out the patient's treatment course. M.L, S.B.H, and M.N collected the data. S.Z and R.S drafted the manuscript. All authors proofread and accepted the final version of the manuscript.

## FUNDING INFORMATION

No financial support was received for this case report.

## COMPETING INTERESTS

The authors declare that they have no competing interests.

## ETHICAL APPROVAL

Written informed consent was obtained from the patient in our study. The purpose of this research was completely explained to the patient and was assured that their information will be kept confidential by the researcher. The present study was approved by the Medical Ethics Committee of the academy.

## CONSENT

Written informed consent was obtained from the patient for publication of this case report and any accompanying images. A copy of the written consent is available for review by the Editor‐in‐Chief of this journal.

## Data Availability

All data regarding this case has been reported in the manuscript. Please contact the corresponding author if you are interested in any further information.
